# Association of Glycative Stress With Motor and Muscle Function

**DOI:** 10.3389/fphys.2022.855358

**Published:** 2022-02-24

**Authors:** Tatsuro Egawa, Tatsuya Hayashi

**Affiliations:** ^1^Laboratory of Health and Exercise Sciences, Graduate School of Human and Environmental Studies, Kyoto University, Kyoto, Japan; ^2^Laboratory of Sports and Exercise Medicine, Graduate School of Human and Environmental Studies, Kyoto University, Kyoto, Japan

**Keywords:** glycation stress, advanced glycation end products, exercise, aging, diabetes, skeletal muscle, sarcopenia, frailty

## Abstract

Glycative stress is a type of biological stress caused by non-enzymatic glycation reactions, which include advanced glycation end product (AGE) formation, AGE accumulation, glycation-driven dysfunction of proteins and cellular signaling, inflammation, oxidation, and tissue damage. Increased glycative stress derived from hyperglycemia and lifestyle disorders is a risk factor in metabolic and age-related diseases, such as type 2 diabetes, cardiovascular disease, cancer, Alzheimer’s disease, osteoporosis, and dementia. Studies have shown that AGE accumulation is correlated with the age-related loss of muscle mass and power output, also called sarcopenia. Mechanistically, dysfunctions of contractile proteins, myogenic capacity, and protein turnover can cause glycative stress-induced skeletal muscle dysfunction. Because the skeletal muscle is the largest metabolic organ in the body, maintaining skeletal muscle health is essential for whole-body health. Increasing awareness and understanding of glycative stress in the skeletal muscle in this review will contribute to the maintenance of better skeletal muscle function.

## Introduction

**FIGURE 1:**
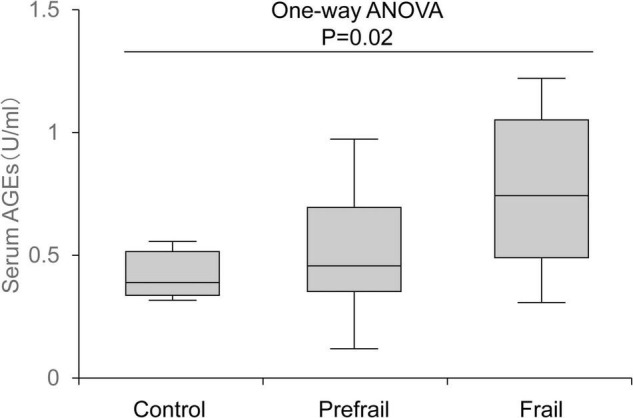
Association between serum advanced glycation end product (AGE) levels and frailty status. AGE level increased according to the degree of frailty. The top and bottom regions of the boxes indicate 75 and 25 percentiles, respectively. The line through the middle of each box represents the median. The upper and lower error bars show minimum and maximum values, respectively (Yabuuchi et al., 2020).

Proteins are functionally regulated through post-translational modifications. “Glycation” is a type of post-translational modification that occurs non-enzymatically and involves a chemical reaction in which proteins bind to sugars and eventually become modified into advanced glycation end products (AGEs). AGE-modified proteins not only have impaired function but also activate inflammatory signaling through the AGE receptor and adversely affect physical function. In addition, glycation occurs not only proteins but also in lipids or nucleic acids, and thereby affecting various biological functions. The harmful effects caused by such glycation are collectively called “glycative stress (or glycation stress).” Because AGE-modified proteins increase with age and are involved in the development of age-related diseases, such as cancer, Alzheimer’s disease, osteoporosis, and diabetic complications, glycative stress is attracting attention as an aging-promoting factor along with oxidative stress. In recent years, its association with the onset of frailty syndrome and sarcopenia has been clarified (Figure 1), and it is being recognized as a risk factor for lowering motor function and muscle function (Yabuuchi et al., 2020). This review outlines the effects of glycative stress on motor function and muscle function as well as the effects of exercise on glycative stress.

## Glycative Stress and Motor Function

Because AGE modification of proteins is an irreversible reaction, they are degraded through protein turnover. However, because protein turnover decreases with aging, the accumulation of AGEs in the body progresses, leading to an increase in glycative stress. Accumulation of AGEs causes various types of decline in biological function. The following reports discuss their effect on motor function.

In a study conducted in the United States, 394 elderly women (≥ 65 years old) were followed-up for 30 months. The results revealed that the level of carboxymethyl lysine (CML), which is a typical AGE, in the blood is a risk factor for poor walking ability (Sun et al., 2012). In addition, cohort studies of elderly people (≥ 65 years old) in Germany and Netherlands have found that blood and subcutaneous levels of AGEs were inversely associated with health-related quality of life (Drenth et al., 2018; Ebert et al., 2019). Negative correlations between blood CML level and walking speed and subcutaneous AGE level and maximal oxygen uptake have also been shown (Semba et al., 2010; Kunimoto et al., 2020). Collectively, the accumulation of AGEs in the body is a risk factor for decreased motor function.

## Glycative Stress and Muscle Function

The decline in motor function, induced by the accumulation of AGEs, is caused by the deterioration of musculoskeletal function. Several reports have shown the effects of glycative stress on skeletal muscle performance.

A cohort study of 559 elderly women (≥ 65 years old) in the United States has found an inverse relationship between blood CML level and grip strength (Dalal et al., 2009). Furthermore, the Nagahama Cohort Study in Japan, which enrolled 9203 middle-aged people (average age 57.8 years), has shown that the more advanced the accumulation of skin AGEs the lower the muscle mass and grip strength (Tabara et al., 2019). In surveys of patients with type 1 diabetes and type 2 diabetes, muscle mass and strength (grip strength and knee extension strength) decreased with AGE accumulation (Mori et al., 2017, 2019). Some factors that cause the AGE-driven deterioration of muscle mass and strength include: (1) decline in muscle contractile activity due to AGE modification of structural proteins, such as myosin, actin, and collagen, (2) decline in myogenic capacity, and (3) suppression of protein synthesis and stimulation of protein breakdown (Supplementary Figure 1^1^).

## Evidence of the Effects of Exercise on Glycative Stress

Because glycative stress increases due to lifestyle disorders, such as lack of exercise, nutritional imbalance, drinking, smoking, and lack of sleep, lifestyle improvement is the basis for reducing glycative stress. The following is an example of how regular exercise improves glycative stress.

**FIGURE 2:**
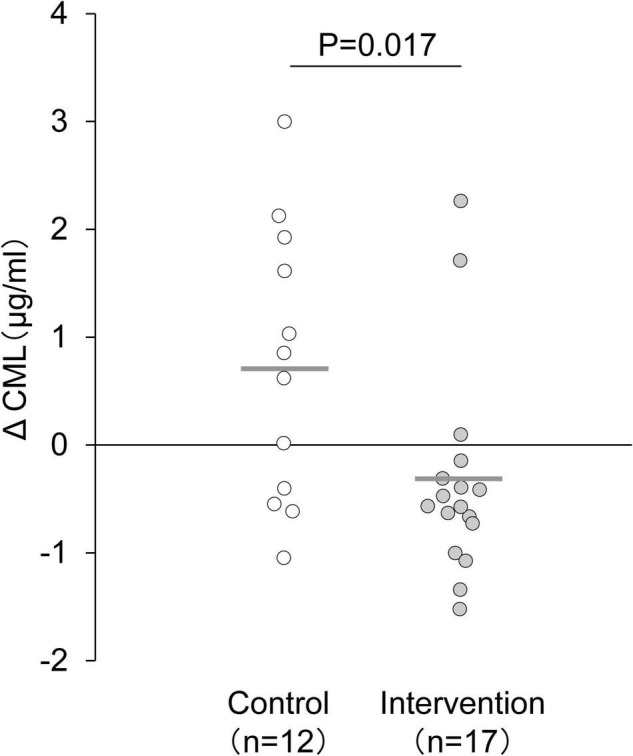
Changes in serum CML level after 12-week exercise intervention. The delta value was calculated as the postintervention value minus the baseline value. The mean decrease in serum CML level was significantly larger in the intervention group than in the control group. Horizontal bars indicate mean values. Changes in CML level between the intervention and control groups were compared using an unpaired *t*-test (Yoshikawa et al., 2009).

An intervention study in 2009, in Japan (Yoshikawa et al., 2009) reported changes in blood AGE levels after exercise intervention in non-diabetic middle-aged women (35–70 years old) who did not exercise. In this study, the participants were randomly assigned into the intervention or non-intervention groups. The intervention group performed 60-min walking per week for 12 weeks at 60% of maximum heart rate (HRmax). As a result, their blood CML level decreased, accompanied by the loss of weight, body mass index, body fat mass, and improvement of blood lipid levels (Figure 2); moreover, their decreased blood CML level correlated with the increased number of steps per day.

Another study reported the effects of resistance training on diabetic patients. An intervention study in Australia, in 2017 examined the changes in blood AGE levels after resistance training for 6 weeks in 17 diabetic patients (27–60 years old) (Russell et al., 2017). The participants conducted 15 types of resistance training exercises, three times a week, for up to 60 min each time at an intensity of 65%–85% of one repetition maximum. As a result, their blood AGE levels were lower than those before training, accompanied by a decrease in body fat mass, increase in lean body mass, and improvement of glucose tolerance and blood lipid levels.

The mechanism by which AGE accumulation is reduced by regular exercise is that the raw materials for glycation reaction, such as sugars and aldehydes, are reduced by improving glucose tolerance and blood lipid levels. It has also been reported that bicycle ergometer exercise training (80% HRmax, 45 min, 3 times/week, 12 weeks) increased the gene expression of enzymes that degrade precursor glycation products (Radom-Aizik et al., 2005). In addition, exercise might enhance AGE degradation by stimulating protein turnover and suppressing oxidation-associated glycation (glycoxidation) by strengthening the antioxidation system.

## Indicator of Glycative Stress

The level of AGE accumulation is a common indicator of the status of glycative stress in the body. Measuring blood AGE levels is the most commonly used method. However, recently, devices for measuring subcutaneous fluorescent AGEs under the skin in a non-invasive manner are developing. In addition, the blood level of the soluble receptor for AGE (sRAGE), which captures AGEs, may also be used as an index of the status of glycative stress. However, there is no consistent evidence as it has been reported that sRAGE increases or decreases with exercise. In a 12-year follow-up study of 1779 participants (45–83 years old), blood AGEs, sRAGE, and the AGE/sRAGE ratio were less correlated with mortality, and highly stable subcutaneous AGEs can be a better indicator (Ebert et al., 2020). Hemoglobin A1c (HbA1c), which is an Amadori product and an intermediate of glycation reaction, may also be an effective indicator of glycative stress in patients with diabetes.

## Conclusion

Preventing age-associated increase in glycative stress is a challenge; however, slowing its rate of increase by improving the lifestyle with regular exercise is possible. Decreased motor function, including that of skeletal muscles, is a major factor that leads to the need for long-term care, and taking care not to accumulate glycative stress is necessary to avoid such a condition. Our recent research showed that there is a negative correlation between subcutaneous AGE levels and leg strength, even in young men (mean age 19 years) (Egawa et al., 2021). Furthermore, glycative stress was reported to affect endurance capacity in children (mean age 7 years) (Kochli et al., 2020). Taken together, the adverse effects of glycative stress on motor function occurs regardless of age. Therefore, it may be important to understand the status of glycative stress from a young age as a precaution against deterioration of motor function and need for long-term care.

## Author’s Note

This is a English language translation of “Glycative stress and motor function (Touka-sutoresu to undou-kinou)” originally published in the Journal of Practical Diabetes, 38 (5), 587–589, 2021. Permission was granted by Ishiyaku Publishers, Inc (https://www.ishiyaku.co.jp/ourco/aboutus.aspx).

## Author Contributions

TE and TH contributed to the conception and design of the review article. TE prepared the manuscript. TH revised the manuscript. Both authors approved the final draft of the manuscript.

## Conflict of Interest

The authors declare that the research was conducted in the absence of any commercial or financial relationships that could be construed as a potential conflict of interest.

## Publisher’s Note

All claims expressed in this article are solely those of the authors and do not necessarily represent those of their affiliated organizations, or those of the publisher, the editors and the reviewers. Any product that may be evaluated in this article, or claim that may be made by its manufacturer, is not guaranteed or endorsed by the publisher.

## Footnotes

1
https://doi.org/10.6084/m9.figshare.19069613
